# Reply to Özdemir, “Measles-Mumps-Rubella Vaccine and COVID-19 Relationship”

**DOI:** 10.1128/mBio.02465-20

**Published:** 2020-09-22

**Authors:** Paul L. Fidel, Mairi C. Noverr

**Affiliations:** a Department of Oral and Craniofacial Biology, Louisiana State University Health – School of Dentistry, New Orleans, Louisiana, USA; b Department of Microbiology and Immunology, Tulane University School of Medicine, New Orleans, Louisiana, USA; Harvard Medical School

## REPLY

First, we thank Dr. Özdemir for having enough interest in our paper (1) to submit the Letter to the Editor. As an Opinion/Hypothesis paper, the very nature of the publication is to spark debate or counterarguments. Our recently funded clinical trial (Parsemus Foundation and Fast Grant, Emergent Ventures at the Mercatus Center, George Mason University) and primate studies (Fast Grant) are still in preliminary stages; therefore, we do not yet have data and associated statistical analyses to support the hypothesis that can be used to fully respond to the points made by Dr. Özdemir. Since our publication, however, additional anecdotal/circumstantial evidence supporting the hypothesis has accumulated with very little negative evidence. This includes information passed on to us by individuals with personal experiences or knowledge otherwise (i.e., military information, refugee camps, countries with recent measles-mumps-rubella [MMR] vaccination campaigns for adults) or via prepublications. But to address the specific points made, we offer the following.

Our opinion manuscript was not intended to be an epidemiological analysis of COVID-19 rates/severity in relation to recent live attenuated vaccine exposure. There are other groups working on these types of analyses with some positive outcomes (2, 3). However, in analyzing data from countries with mandatory BCG vaccination campaigns, it is essential to correlate recent BCG exposure with COVID-19 rates and severity. While trained innate immunity can be long-lived, it is certainly not lifelong and would be expected to wane after exposure to the vaccine as a child. It has been suggested that epidemiological data need to be reanalyzed by correlating functional immunity to BCG (correlates to more recent vaccine administration), but these analyses have yet to be published. In published work, however, there are just as many examples of countries with recent BCG vaccinations showing low COVID-related mortality (2).

We want to stress as well that the concept most widely circulated with BCG vaccination relative to COVID-19 is an immune-enhancing trained innate immunity. Our hypothesis for live attenuated vaccines (i.e., MMR), based on our animal studies of fungal/bacterial sepsis (4), is one of immune tolerance through the induction of the myeloid-derived suppressor cells (MDSCs) that inhibit/dampen sepsis associated with COVID-19. Of course, genetic attributes and comorbidities will influence susceptibility and sequelae associated with COVID-19 infection, but even if the live attenuated MMR vaccine invoked weaker but measurable protection against COVID-19 in certain groups of individuals, this still equates to a low-risk, high-reward strategy. There is also the possibility that the effects may be enhanced if one or more components of the MMR vaccine have cross-reactivity to COVID-19 (5, 6) such that the benefits would range from reduction/dampening of sepsis to some level of direct immunity against COVID-19. On the other hand, we made the argument in our paper, but will repeat it here, that seasonal flu is not associated with inducing acute sepsis. Therefore, if our theory is correct, live attenuated vaccines that induce the MDSCs would have little effect on seasonal flu.

The question as to whether there may be a cumulative effect of live attenuated vaccines for the nonspecific beneficial immune effects is interesting. While the current clinical trials are not investigating this issue directly, we have focused on the MMR vaccine as it is widely available and has the potential for any or all of the three components to induce the MDSCs. However, based on our data in the animal model of fungal/bacterial sepsis, very strong long-lasting protection is afforded from one administration of the attenuated fungal isolate (7). Hence, cumulative effects may not be important with the exception of extending the time for optimal protection with timely boosters.

Finally, while it is true that we do not know how long the trained innate immunity persists, the randomized clinical trial of MMR versus placebo in health care workers and the nonhuman primate study that will test MMR or BCG in a model of COVID-19 infection will go far to answer these questions. To date, the trained innate response with BCG suggests the immunity is functional for approximately 1 year based on infant vaccinations (8). The studies in the animal model of fungal/bacterial sepsis show that the MDSCs are induced quickly (within 7 days of vaccination) and are relatively long-lived (up to 120 days) (7, 9). Data also show that the protection afforded by the trained innate response can be achieved with multiple lethal challenges over time (9). Since these are mouse studies with relatively short lifespans, we can predict a fairly long half-life for the immune-tolerant trained response in humans. Indeed, the sequelae and hospitalization associated with COVID-19 infection are much reduced in children, teenagers, and even young adults in their early 20s (10, 11). Children often get the MMR vaccine up to three times by age 11. Thus, it is possible that the nonspecific protective effects provided by the live attenuated vaccines are quite long-lived. Until more is known, however, we are suggesting that the MMR vaccination be given to eligible adults who have not had the vaccination within a year, as an immune preventive against the worst sequelae of any subsequent COVID-19 infection.
